# Distraction by deviant sounds: Response repetition vs. response change

**DOI:** 10.1371/journal.pone.0357782

**Published:** 2026-09-11

**Authors:** Elena García-López, Fabrice B. R. Parmentier

**Affiliations:** 1 Department of Psychology & Research Institute of Health Sciences (iUNICS), University of the Balearic Islands, Palma, Spain; 2 Balearic Islands Health Research Institute (IdISBa), Palma, Spain; 3 School of Psychological Science, University of Western Australia, Perth, Australia; University of Hamburg, GERMANY

## Abstract

In oddball paradigms, rare deviant stimuli reliably produce behavioral distraction, disrupting performance in ongoing primary tasks. However, the specific mechanisms through which deviance affects response selection and execution remain unclear. Recent evidence suggests that deviant sounds may differentially affect response repetitions and response changes, but whether these effects reflect a single underlying process or functionally independent mechanisms has not been established. We investigated this question using a cross-modal auditory-visual oddball task in which participants (*N* = 115) identified visual target locations while ignoring standard (80%) or deviant (20%) tones. Critically, we manipulated response set size between participants (two-alternative forced-choice [2-AFC] vs. four-alternative forced-choice [4-AFC]) while holding deviant probability constant, testing whether deviance effects on repetitions and changes differ in their sensitivity to response uncertainty. Results revealed a clear dissociation: deviant sounds slowed response repetitions in both conditions, demonstrating response-specific inhibition that was insensitive to response uncertainty, but facilitated response changes more strongly in 2-AFC than 4-AFC, demonstrating context-sensitive facilitation that scales inversely with the number of response alternatives. These findings suggest that deviance effects on response repetition and response change may reflect functionally independent mechanisms with different computational properties, and that unexpected events may shape action control through coordinated inhibitory and facilitative processes.

## Introduction

Unexpected events often demand rapid behavioral adaptation. While ignoring extraneous stimuli usually optimizes performance, completely blocking external input can be maladaptive, since sudden changes may signal important shifts in environmental contingencies. For example, for a person sitting in a library and engrossed in a book, focusing attention on the text while remaining oblivious to surrounding noise may seem like an efficient way to complete the task, but the ability to detect a sudden fire alarm remains crucial from an adaptive perspective. Abundant research has demonstrated the existence of neurocognitive mechanisms that respond to unexpected sounds and lead to an orienting response toward such stimuli [1–9]. These adaptive mechanisms, however, can also produce deviance distraction when the deviant stimulus is to be ignored [e.g., 2].

### Deviance distraction: Behavioral evidence

Since Schröger's [3] seminal work, evidence has accumulated showing that deviant stimuli trigger specific electrophysiological signatures [1,3,10] and lengthen response times in primary tasks [2,3,11]. Furthermore, this deviance distraction (distraction induced by the presentation of a deviant stimulus) is not modality-specific: it occurs within audition [3,12] as well as across distinct modalities [1,13–15]. Nor is deviance distraction task-specific, having been reported in categorization [e.g., 1, 16–18], stimulus change detection [19], go/no-go [20,21], visual matching, serial recall [22–25], gap detection [26], or duration discrimination tasks [27,28]. Crucially, deviance distraction reflects violations of sensory predictions rather than stimulus rarity [29–32]. Indeed, it is reduced or even eliminated when sounds become predictable [30,33,34], but it appears to be immune to the predictability of one's own actions [14]. Predictions are not only based on recent past experience of the to-be-ignored stimuli but also on their integration with the environmental context, such as task-irrelevant background visual [35] or auditory [36] stimuli.

Of particular interest, some have suggested that an unexpected change in our immediate environment impacts motor behavior by first temporarily freezing it [8,37–41] and disrupting ongoing actions and/or biasing the system toward initiating a new one [42–45]. From an adaptive standpoint, unexpected stimuli trigger an orienting response [6,7] and enable the organism to react to events violating predictions [46] in the form of unexpected stimuli or the unexpected outcome of one's own actions [45,47,48]. Deviant stimuli trigger a re-evaluation of current models of the environment [49,50]. They also elicit rapid, global inhibition of motor actions [51,52], which was recently shown to also affect eye movements during reading and scanning tasks [37–40].

### Response repetition vs. change in the oddball paradigm

Interestingly, and somewhat overlooked, some evidence suggests that behavioral deviance distraction is greater for response repetitions compared to response switches in two-alternative forced-choice (2-AFC) oddball tasks [43], though similar event-related potentials (MMN, P3a, RON) were observed in these two conditions. This suggests that response sequences modulate behavioral outcomes but do not eliminate underlying deviance distraction mechanisms. Building on this finding, Roeber et al. [44] made a critical advance by systematically manipulating both the global probability of deviant stimuli (rare vs. frequent) and the local sequential structure of trials. Using a 4-to-2 stimulus-response mapping (four syllables mapped onto two responses), they demonstrated that deviance distraction effects varied as a function of the type of transition between successive trials. Specifically, they found that the largest behavioral deviance distraction effects occurred for trials in which both stimulus and response repeated (identical syllables), while trials requiring response changes showed smaller or absent deviance distraction. Crucially, they found that these effects differed between rare deviants (12.5% of trials) and frequent pitch changes (50% of trials): rare pitch changes produced greater deviance distraction for response repetitions and reduced the syllable repetition benefit more than frequent pitch changes. Moreover, they found that the response change benefit (the advantage for switching responses when the stimulus changed) was smaller for rare deviants than for frequent changes. These findings led Roeber et al. [44] to conclude that deviance distraction effects cannot be fully explained by sequential effects alone, and that rare, unexpected changes engage specialized processing mechanisms. They attributed their findings to a “motor bias toward change” [53,54], whereby any stimulus change creates a tendency to switch responses, which benefits trials requiring response changes but interferes with trials requiring response repetitions. Despite this advance, Roeber et al.’s [44] design could not determine whether the effects of deviance on repetitions and changes reflect a single underlying process or two independent mechanisms, because their manipulation of deviant probability affected both response types simultaneously.

We recently reported a study akin to that of Roeber et al. [43] where we carefully controlled the stimulus sequences to balance the proportions of trials requiring response repetitions and switches [42]. Our data revealed a large deviance distraction effect (slower responses) for response repetitions and a significant facilitation (faster responses) for response switches (results compatible with those of Roeber et al. [43]). These results can be interpreted in two non-mutually exclusive ways. One possibility is that unexpected changes in the task environment bias the cognitive system toward switching responses. This “bias to switch” heuristic is present in the literature [54,55] and was cited by Roeber et al. [43,44] to explain their results. This interpretation certainly seems appealing and fits well with the general observation that the cognitive system extracts stimulus contingencies to economize resources and prepare motor responses. Such a bias toward changing responses would be especially advantageous in 2-AFC tasks where rejecting response R1 automatically means adopting response R2. A second, non-mutually exclusive, possibility is that unexpected stimuli inherently hinder response repetition. From an adaptive standpoint, organisms may inhibit the repetition of a specific action when environmental circumstances change. This is analogous to what can be observed in the behavior of a rodent foraging for food. If surrounding conditions remain stable, foraging goes uninterrupted. However, the detection of a sudden and unexpected noise in nearby bushes marks a change in context in which foraging may no longer be the appropriate response and should therefore be inhibited, resulting in a typical freezing reaction by the rodent. The notion of a response cost under changing circumstances is supported by the task-switching literature, which consistently shows slower switch trials than repetition trials [53,56–58] and fits the general notion of a stopping mechanism in the face of an unexpected event [59].

A key question is whether these two effects (the hindering of response repetitions and the facilitation of response changes) reflect a single unified process or two functionally independent mechanisms. While Roeber et al.’s [44] study provided important evidence that deviance distraction cannot be reduced to sequential effects, it did so varying the number of stimuli while maintaining a fixed response set size. The present study adopts a complementary approach. Rather than manipulating deviant probability, we held it constant (always rare, as in typical deviance distraction paradigms) while systematically varying response set size (2-AFC vs. 4-AFC).

Under a repetition-inhibition hypothesis, deviant sounds should selectively suppress the previously executed response independently of the number of alternatives. If this is the primary mechanism at play, we should observe equivalent deviance distraction effects across response set sizes for response repetitions: deviant sounds should slow response repetitions equally in 2-AFC and 4-AFC tasks because the inhibition targets the specific prior response, regardless of how many alternatives exist. In contrast, under a *response-switch facilitation hypothesis*, deviant sounds should facilitate the selection and execution of an alternative response (i.e., a response other than that produced on the previous trial), and this facilitation should vary with response set size. Specifically, we would expect stronger facilitation in a 2-AFC task, where switching away from R1 necessarily means adopting a specific alternative R2, than in a 4-AFC task where the bias to change responses must be distributed among multiple alternatives (R2, R3, and R4). This prediction follows from well-established principles in choice reaction time research: response uncertainty (the a priori uncertainty about which response will be required on a given trial, operationalized here as response set size) increases with the number of equiprobable alternatives [60,61], and activation spread across multiple alternatives should weaken the facilitation of any single response. Critically, if deviant sounds exert both repetition inhibition and switch facilitation effects independently, we should observe a dissociation: deviance distraction for response repetitions should be immune to response set size (supporting repetition inhibition), while facilitation for response changes should be modulated by response set size (supporting switch facilitation). Note that, because the two effects are expected to differ in their sensitivity to response set size rather than to share a common modulation, this prediction is tested most directly through the two targeted planned contrasts reported below rather than through an omnibus three-way (Response Type × Sound Condition × Response Set Size) interaction, which we therefore did not expect to reach significance. Such a pattern would establish that deviance distraction reflects the coordinated operation of two dissociable processes — some relatively automatic (global motor inhibition), and others more strategic (biased response selection) — and would provide a more process-specific account than the general “motor bias toward change” explanation.

## Method

### Participants

A total of 118 participants were recruited. Because our task critically relied on lateralized motor responses to distinguish between key experimental conditions, we tested only right-handed participants. Our central hypothesis (that deviant sounds trigger the inhibition of one response and the potentiation of the alternative) presupposes that these competing responses are strongly lateralized at the cortical level. In a task in which one lateralized response must be adopted while the other is inhibited, data from left-handed participants would introduce noise and jeopardize the task's sensitivity, because the more bilateral cortical organization typical of left-handed individuals translates into more similar activity on either side of the brain (including the motor cortex) regardless of the response made [62–66]. Two were excluded for not following the task instructions and one due to a technical problem during the experiment. The final sample therefore included 115 right-handed participants: 95 females (*M age* = 20.3 years, SD = 4.7) and 20 males (*M age* = 21.0 years, SD = 3.0). None of the participants reported any auditory, neurological, or psychiatric condition. All participants had normal hearing and normal or corrected-to-normal vision. All participants provided written informed consent prior to taking part in the study and received either course credit or a small monetary compensation in exchange for their participation. The study was conducted in adherence with the Declaration of Helsinki and approved by the Ethics Committee of the University of the Balearic Islands (319CER23). The data were collected between November 29, 2023, and January 24, 2024.

### Stimuli and procedure

All participants performed the cross-modal auditory-visual oddball task in a sound-attenuated booth. The task was designed using E-Prime 3.0 software (Psychology Software Tools, Pittsburgh, PA). Responses were recorded using a computer keyboard. The audio stimuli were mono MP3 files (44.1 kHz) and were presented diotically through headphones at an intensity of approximately 70 dB SPL.

#### Visual stimuli.

**Fig 1:**
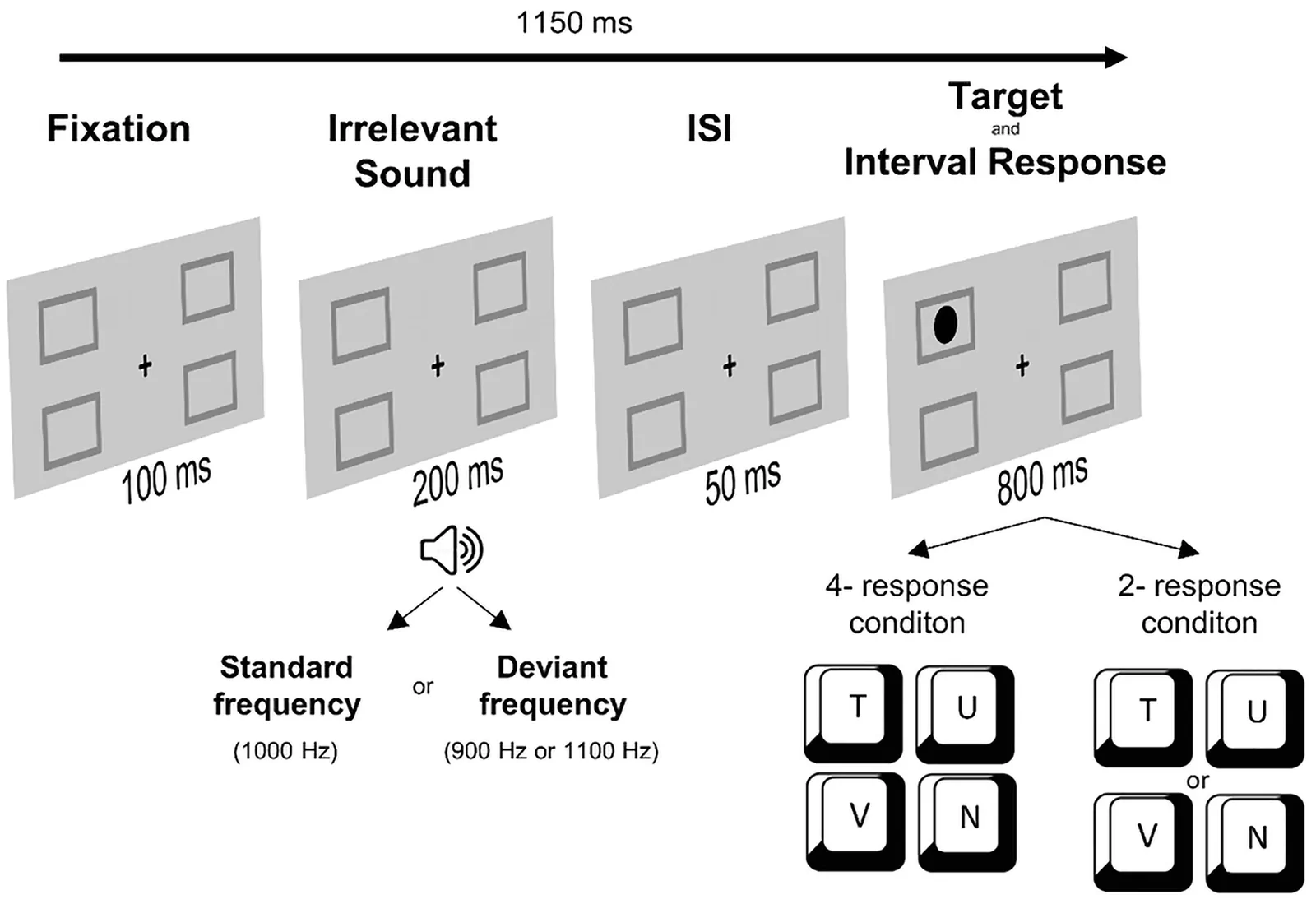
Schematic representation of the experimental paradigm. This figure illustrates the different phases of a single trial and the two response set size conditions (presented between-participants).

In each trial, participants were required to identify the location of a visual target (a black dot with a diameter of approximately 2.7°; HEX color: #010101) on a gray screen (HEX color: #c0c0c0). The target could appear in one of four possible locations within a 2 × 2 configuration (upper-left, lower-left, upper-right, and lower-right position). Each location was marked by a dark gray rectangle (size 5.8° × 4.34°, border thickness 0.82°, HEX color: #808080; see Fig 1). Each rectangle was located 11.02° from the central fixation point (distance measured from the center of the screen to the center of each rectangle). Across trials, the target appeared equiprobably in each of the four locations in a quasi-random fashion. The appearance of the target in a specific location was equally often followed by its presentation in each of the four locations (that is, the location of a target was entirely nonpredictive of its location in the next trial).

#### Auditory stimuli.

Before each target appeared, a to-be-ignored sound was played. Three different sounds were used in the experiment: a standard tone (1000 Hz sinewave, 80% of trials) and two deviant tones (900 Hz and 1100 Hz sinewaves, 10% of trials each). All sounds lasted 200 ms, were normalized, and had 10 ms fade-in and fade-out ramps. The three sounds and four target locations were orthogonally crossed (such that all sound-location pairings occurred with equal probability across the task). The sound sequence was quasi-random with the following constraints: the deviant tones (900 Hz and 1100 Hz) were used 16 times each (i.e., 32 deviant trials in total) within each block of 160 trials and were never presented in immediate succession.

#### Response mapping.

Participants were instructed to respond based on the location of the visual target while ignoring the preceding sound. They were provided with either two or four response keys depending on the experimental condition (between-participants). In the 2-AFC set size condition, participants used “t” and “u” or “v” and “n” (counterbalanced across participants) to indicate whether the target appeared on the left or right side of the screen (“t” or “v” for the left vs. “u” or “n” for the right, respectively). In the 4-AFC set size condition, participants used all four keys (“t,” “v,” “u,” “n”) to indicate the exact target location, with each key corresponding to one of the four positions: “t” for upper-left, “v” for lower-left, “u” for upper-right, and “n” for lower-right. This keyboard layout mirrored the 2 × 2 spatial configuration shown on screen. This method ensured that participants in both conditions were presented with stimuli that had identical characteristics.

#### Trial and task structure.

The test phase consisted of 960 trials divided into six blocks of 160 trials each. Participants were allowed a short break between blocks if they wished. Each trial lasted a total of 1150 ms and followed a fixed sequence: a fixation point was presented for 100 ms, during which the four framed locations were visible on the screen. These visual elements remained visible throughout the trial. The fixation point was followed by the presentation of a sound lasting 200 ms. The sound’s offset was followed by a further 50 ms delay before the visual target appeared and remained on screen for 800 ms, which was also the time window available to participants to respond.

Before beginning the test phase, participants completed two practice blocks consisting of 20 trials each (16 standard trials and 4 deviant trials). The timing of the practice trials was identical to that of the test trials. The first practice block included a 1000 ms feedback screen after each trial, indicating whether the response was correct (shown in green text, HEX color: #008000), incorrect (in red, HEX: #ff0000), or absent (in white, HEX: #ffffff). The second practice block did not include feedback and was intended to help participants adjust to the timing and pace of the test phase.

### Statistical analysis and planned comparisons

We analyzed the mean proportion of correct responses and the mean response times (RTs) for correct responses. RTs were measured from the moment of presentation of the target stimulus. Effect sizes are reported as partial eta-squared values for F tests, and as Cohen’s d_z_ for within-participant comparisons and d for between-participants comparisons [67], both reported as *d* hereafter for simplicity. In addition to frequentist statistics, we also report the Bayes factor (BF_10_) to assess the credibility of the experimental hypothesis relative to that of the null hypothesis given the data. For each interaction, we report the Bayes factor obtained by directly comparing two nested models: a baseline model including all relevant lower-order terms and the same model with the interaction term added. This quantifies the evidence that the interaction improves model fit beyond the baseline model. Values below 1/3 are considered as substantial to strong support for the null effect, while values above 3 are regarded as substantial to strong support for the presence of an effect [68,69]. When the outcomes of the frequentist and Bayesian analyses diverged, we adopted a conservative interpretation; that is, we treated the evidence as inconclusive. An a priori power analysis was carried out based on the most critical hypothetical effect our experiment was testing: the 2 (deviant vs. standard) × 2 (response set size 2 vs. 4) interaction for response switches. Since the number of alternative responses changes from one (totally determined) to three (1/3 probability per response), we thought it reasonable to plan for a medium effect size for the key interaction above (f = .25). For a Type I error probability of .05 and a power of .95, the minimum total sample size is 54 (27 per group). Our recruited sample size was larger (N = 115: 57 in the 2-AFC condition, 58 in the 4-AFC condition).

To compare like with like across the 2- and 4-AFC conditions, we retained only trials in which the stimulus and the response either both repeated or both changed relative to the previous trial. In the 4-AFC condition, the one-to-one mapping between stimuli and responses made this automatic: a repeated response necessarily coincided with a repeated stimulus, and a changed response with a changed stimulus. In the 2-AFC condition, by contrast, the two-to-one mapping meant that a response repetition could occur even when the stimulus changed; we therefore excluded those stimulus-change response-repetition trials. Because response repetition versus response change can only be meaningfully defined relative to the preceding trial, the first test trial was discarded from the analyses. For the purpose of trial balancing, this first trial had been formally assigned to the response-change condition, so its exclusion reduced the number of analyzable response-change trials by one. Thus, out of the 960 test trials, 959 entered the analyses. In the 4-AFC condition, the nominal design included 96 deviant and 384 standard trials in both the response-change and response-repetition conditions, with one response-change trial excluded because it was the first test trial. In the 2-AFC condition, we retained the response-change trials, minus this first trial, as well as the response-repetition trials in which the stimulus repeated, comprising 48 deviant and 192 standard trials. The response-repetition trials involving a stimulus change, comprising 48 deviant and 192 standard trials, were discarded. Trials with response times shorter than 60 ms, which are too short to reflect a stimulus-driven response [70,71], were treated as anticipations and excluded from the analysis, as were the first standard trials following a deviant trial [since these have been demonstrated to contain residual distraction, [16,17,72]. Finally, to ensure that the type of response (repetition vs. change) provided by participants matched the required response type (repetition vs. change), we excluded post-error trials. Filtering anticipations, post-deviant trials, and post-error trials resulted in the exclusion of 22% and 23% of the trials in the 2- and 4-AFC conditions, respectively.

Hit rates and mean response times for correct responses were analyzed using 2 (deviant versus standard frequency) × 2 (change versus repetition response) × 2 (2- versus 4-AFC set size) mixed ANOVA. While this omnibus analysis characterizes the overall structure of the data, our key theoretical predictions concern the effect of response set size on each deviance effect separately and are therefore evaluated through the planned contrasts described below; under our dual-process account, these dissociable and differentially modulated effects are not expected to produce a significant three-way interaction.

### Critical planned contrasts

Central to testing our key hypotheses, we carried out planned comparisons to test specific a priori predictions: we compared the 2- and 4-AFC conditions with respect to distraction (deviant vs. standard) in the response repetition condition and, separately, in the response change condition. The first contrast served to test the repetition-inhibition hypothesis, namely that if deviant sounds disrupt the mere repetition of a response, the deviant-induced distraction should be insensitive to the response set size. The second contrast tested the response-switch facilitation hypothesis: if deviant sounds facilitate response change, this effect should be stronger when only one response alternative (2-AFC condition) is available than when there are three alternatives (4-AFC condition). For these planned comparisons, we used one-tailed tests.

## Results

### Proportion of correct responses

**Fig 2:**
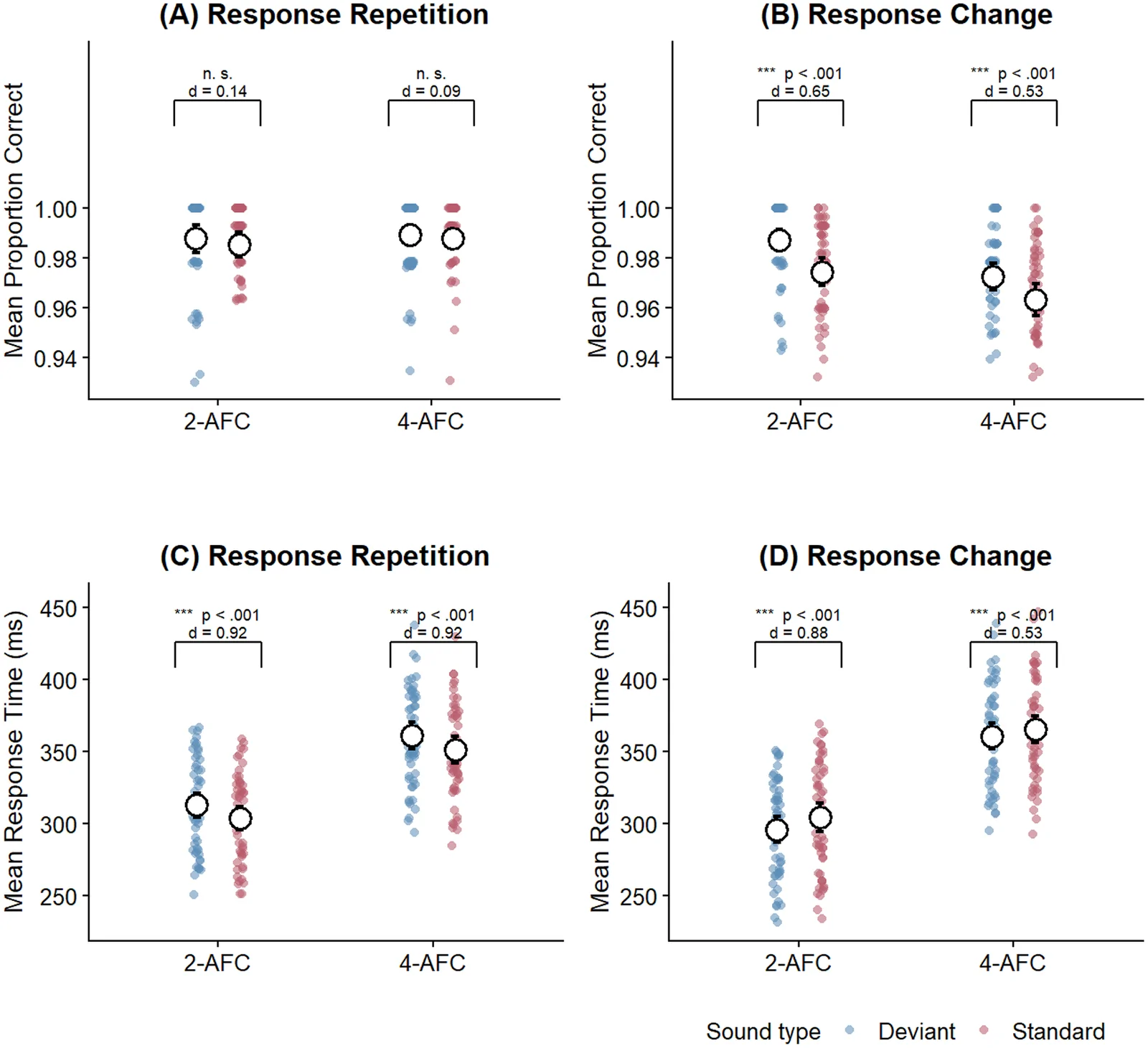
Mean proportion correct (A, B) and mean response time (C, D) for Deviant (blue) and Standard (red) sounds, shown separately for Response Repetition (A, C) and Response Change (B, D) trials, and for the 2-AFC and 4-AFC conditions. Individual data points represent participant means. Open circles denote the group mean; error bars indicate 95% confidence intervals. Brackets indicate the outcome of paired-samples t-tests comparing Deviant and Standard sounds within each response set size condition. *** p < .001; n.s. = non-significant. d = Cohen's d.

Accuracy was near ceiling overall (*M* = .976, *SD* = .018; see Fig 2, panels A & B). The ANOVA revealed a significant main effect of Response Type, *F*(1, 113) = 82.358, *MSE* < .001, *p* < .001, η²*p* = .422, BF10 = 2.341 × 10^10^, with higher accuracy for response repetitions (*M* = .987, *SD* = .015) than response changes (*M* = .972, *SD* = .021). A significant main effect of Sound Condition was also observed, *F*(1, 113) = 35.114, *MSE* < .001, *p* < .001, η²*p* = .237, *BF*_*10*_ = 11465.584, with slightly higher accuracy on deviant trials (*M* = .982, *SD* = .017) than standard trials (*M* = .974, *SD* = .019). Response Set Size did not yield a significant main effect, *F*(1, 113) = 3.779, *MSE* = .003, p = .054, η²p = .032, *BF*_*10*_ = 1.173. Two significant two-way interactions were observed. A Response Type × Response Set Size interaction, *F*(1, 113) = 26.498, *MSE* < .001, *p* < .001, η²*p* = .190, *BF*_*10*_ = 10549.285, reflected lower accuracy for response changes in the 4-AFC condition (*M* = .966, *SD* = .022) than in the 2-AFC condition (*M* = .978, *SD* = .018), with no corresponding difference for response repetitions. A Response Type × Sound Condition interaction, *F*(1, 113) = 14.750, *MSE* < .001, *p* < .001, η²*p* = .115, *BF*_*10*_ = 555.314, reflected greater accuracy for deviant than standard trials specifically for response changes (*M* = .980 vs. .969), with no such difference for response repetitions (*M* = .988 vs. .986). The Response Set Size × Sound Condition and three-way interactions were not significant (both *BF*_*10*_ < 0.23).

Planned comparisons focused on the deviant–standard accuracy difference for each response type. For response changes, accuracy was reliably higher on deviant than standard trials, *t*(114) = −6.326, *p* < .001, *d* = −0.590, *BF*_*10*_ = 2.031 × 10⁶, confirming a facilitation effect. However, unlike the pattern observed for response times (see below), this facilitation did not differ significantly between the 2-AFC (*M* = −.013, *SD* = .020) and 4-AFC (*M* = −.009, *SD* = .018) conditions, *t*(113) = −0.945, *p* = .173, *d* = −0.176, *BF*_*10*_ = 0.482. For response repe*t*itions, the deviant–standard difference was not significant, *t*(114) = 1.246, *p* = .215, *d* = 0.116, *BF*_*10*_ = 0.220, and planned comparisons confirmed *t*his null was consistent across both set sizes, *t*(113) = −0.383, *p* = .703, *d* = −0.071, *BF*_*10*_ = 0.212. The absence of a se*t*-size effect in accuracy for response changes likely reflects a sensitivity limitation: with overall accuracy above 97%, there is little variance available to detect a modulation of a small facilitation effect. We therefore treat response times as the primary measure and return to the set-size effect in that context.

### Response times

The ANOVA conducted on mean reaction times (RTs, Fig 2, panels C & D) revealed no significant effect of Response Type, *F*(1,113) = 0.255, *MSE* = 225.159, *p* = .614, *η²_p_* = .002, *BF*_*10*_ = 0.203. A significant main effect of Sound Condition was observed, *F*(1,113) = 5.602, *MSE* = 47.027, *p* = .020, η² *_p_* = .047, with shorter RTs on standard trials (*M = 331, SD = 44*) than on deviant trials (*M = 333, SD = 45*), but this difference was not supported by the Bayes factor, which was inconclusive (*BF*_*10*_ = 0.499). There was also a significant main effect of Response Set Size, *F*(1,113) = 82.928, MSE = 4241.510, *p* < .001, *η²*_*p*_ = .423, BF_10_ = 5.970 × 10^11^, indicating longer RTs in the four-response set size condition (*M = 360, SD = 34*) compared to the two-response set size condition (M = 302, SD = 33).

A significant interaction effect was observed between Response Type and Response Set Size, *F*(1,113) = 28.792, *MSE* = 225.159, *p* < .001, η² _p_ = .203*, BF*_*10*_ = 3.245 × 10^4^. Further analysis revealed that, in the four-response set size task, RTs were significantly shorter for response repetitions (*M = 353, SD = 34*) compared to response changes (*M = 363, SD = 35*); *t*(57) = 4.710, *p* < .001, *d* = 0.618 (95% CI: .335 *t*o .897), *BF*_*10*_ = 1172.149. In contrast, the opposite effect occurred in the two-response set size task: RTs were shorter when a response change was required (M = 300, SD = 36) compared to a response repetition (*M = 305, SD = 30*); *t*(56) = −2.396, *p* = .020, *d* = −0.317 (95% CI: −.582 to −.050), *t*hough the difference was not conclusive based on Bayesian statistics (*BF*_*10*_ = 1.982).

A significant Response Type × Sound Condition interaction was observed, *F*(1,113) = 155.181, *MSE* = 47.010, *p* < .001, η² _p_ = .579, *BF*_*10*_ = 2.772 × 10^26^. In trials involving a response repetition, RTs increased following a deviant sound (M = 337, SD = 41) compared to the standard condition (M = 328, SD = 40); *t*(114) = 9.875, *p* < .001, *d* = 0.921 (95% CI: .701 *t*o 1.138), *BF*_*10*_ = 1.076 × 10^14^. In contrast, the deviant sound led to shorter RTs in the response change condition (M = 329, SD = 47) relative to the standard condition (M = 335, SD = 47); *t*(114) = −7.528, *p* < .001, *d* = −.702 (95% CI: −.905 to −.497), *BF*_*10*_ = 6.394 × 10^8^. The Response Se*t* Size × Sound Condition and three-way interactions were not significant: *F*(1,113) = 3.284, *MSE* = 47.027, *p* = .073, η² _p_ = .028, *BF*_*10*_ = 0.347; and *F*(1,113) = 1.135, *MSE* = 47.010, *p* = .289, η² _p_ = .010, *BF*_*10*_ = 0.373, respectively. As discussed earlier, this absence of a significant three-way interaction was expected under our dual-process account. If the two effects (repetition inhibition and switch facilitation) operate through independent mechanisms that are simply differently sensitive to response uncertainty, there is no statistical reason to expect a single omnibus three-way interaction term to capture them. Rather, each mechanism is best revealed through its own planned contrast, which tests the theoretically specific predictions directly (see next section).

### Critical planned comparisons

**Fig 3:**
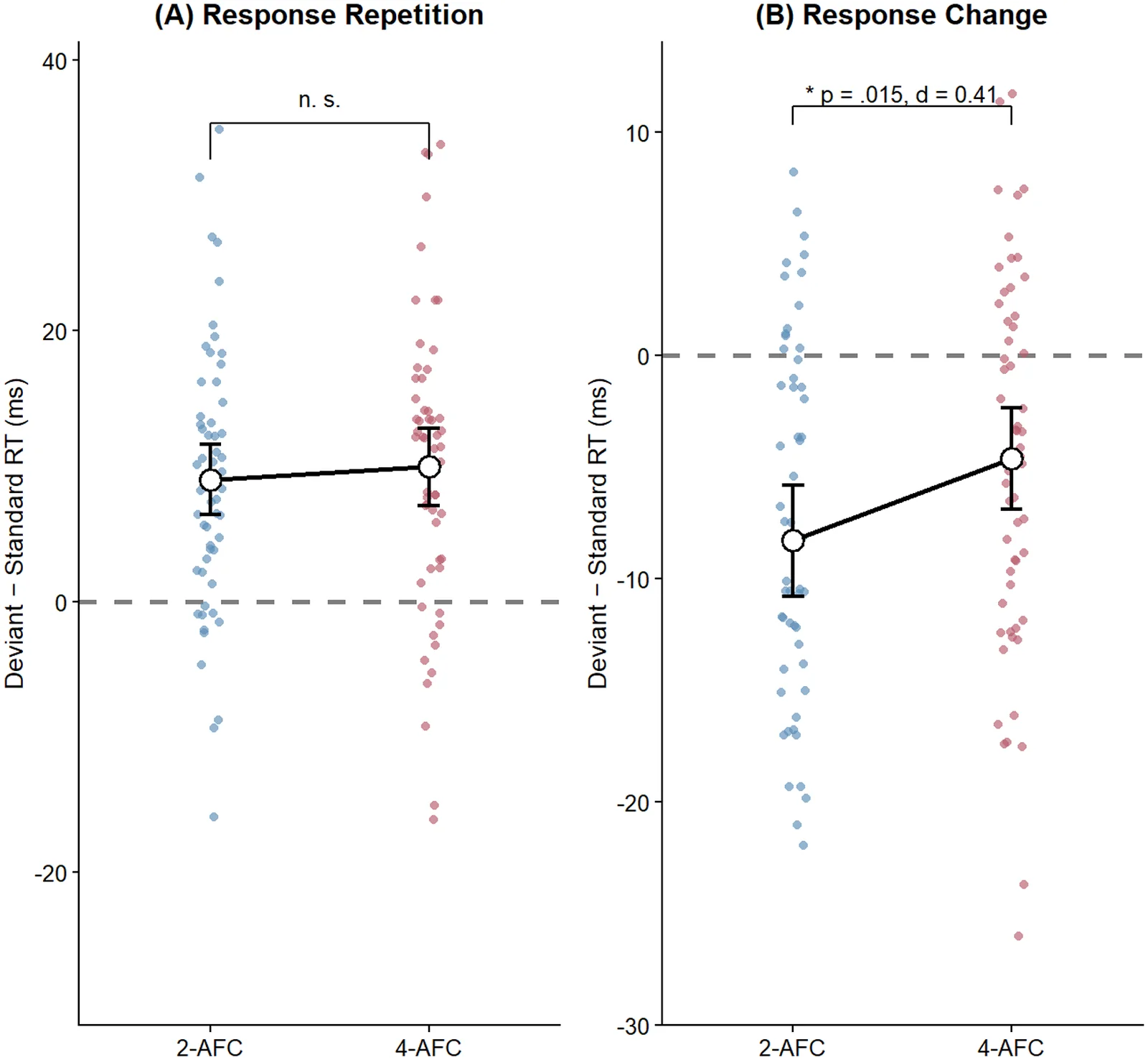
Mean difference in response time between Deviant and Standard trials (Deviant minus Standard, in ms) as a function of response set size (2-AFC vs. 4-AFC) for Response Repetition (A) and Response Change (B) trials. Individual data points represent participant means. Open circles denote the group mean; error bars indicate 95% confidence intervals. The thick line connecting the means aids visual comparison across response set size conditions. Brackets indicate the outcome of paired-samples t-tests comparing the 2-AFC and 4-AFC conditions. n.s. = non-significant.

Most critically, for response change trials, the deviant–standard RT difference was significantly below zero (*M = −6, SD = 9*), *t*(114) = −7.528, *p* < .001, *d* = −0.702 (95% CI: −.905 to −.497), *BF*_*10*_ = 6.394 × 10^8^, indicating faster responses to deviants than to standards. As illustrated in Fig 3, the facilitation induced by deviant sounds for changed responses was significantly greater in the two-response set size condition (*M = −8, SD = 9*) than in the four-response set size condition (*M = −5, SD = 9*), *t*(113) = −2.188, *p* = .015, *d* = −0.408 (95% CI: .097 *t*o ∞), *BF*_*10*_ = 3.269. For response repetition trials, the deviant–standard RT difference was positive (*M = 9, SD = 10*), *t*(114) = 9.875, *p* < .001, *d* = 0.921 (95% CI: 0.701 *t*o 1.138), *BF*_*10*_ = 1.076 × 10^14^, indicating slower responses to deviants than to standards. Our planned comparisons indicated that the deviance distraction effect did not vary with set size: 2-AFC (M = 9, SD = 10) and 4-AFC conditions (*M = 10, SD = 11*); *t*(113) = −0.496, *p* = .621, *d* = −0.092 (95% CI: −.458 to .273), *BF*_*10*_ = 0.221.

In summary, the data show that deviant sounds induced deviance distraction (in RTs) for response repetitions irrespective of the response set size, while they facilitated response change (observed for both the proportion of correct responses and RTs), and this facilitation increased as response set size decreased.

### The impact of stimulus repetition vs. change on response repetitions in the 2-AFC

One unique aspect of our 2-AFC condition is that by using the same set of four stimulus locations, response repetitions could either be accompanied by the repetition of the stimulus (e.g., upper left stimulus followed by upper left stimulus) or by a different stimulus (e.g., upper-left stimulus followed by lower-left stimulus). This distinction did not occur in the 4-AFC (where upper- and lower-left stimuli required different responses). As a complementary analysis, we examined whether a change in stimulus modulated performance in response repetitions within the 2-AFC condition.

The analysis of the mean proportion of correct responses revealed that performance was better for stimulus repetitions (M = .986, SD = .018) than for stimulus changes (M = .965, SD = .025), *F*(1,56) = 74.992, *MSE* < .001, *p* < .001, η² _p_ = .572, *BF*_*10*_ = 1.036 × 10^9^. There was no significant effect of Sound Condition, *F*(1,56) = 0.009, *MSE* < .001, *p* = .926, η² _p_ < .001, *BF*_*10*_ = 0.169, or two-way interaction, *F*(1,56) = 1.295, *MSE* < .001, *p* = .260, η² _p_ = .023, *BF*_*10*_ = 0.396.

The analysis of the mean RTs revealed longer RTs for stimulus change (M = 313, SD = 34) than for stimulus repetition (M = 306, SD = 30), *F*(1,56) = 14.236, *MSE* = 128.068, *p* < .001, η² _p_ = .203, *BF*_*10*_ = 80.014. RTs were longer in the deviant (M = 314, SD = 32) than in the standard (M = 308, SD = 31) condition, *F*(1,56) = 28.290, *MSE* = 73.993, *p* < .001, η² _p_ = .336, *BF*_*10*_ = 6910.297. Of interest, a significant interaction was also observed (*F*(1,56) = 13.248, *MSE* = 37.206, *p* < .001, η² _p_ = .191, *BF*_*10*_ = 43.214), whereby deviance distraction (RT_deviant_-RT_standard_) was greater in the stimulus repetition (M = 9, SD = 10) than in the stimulus change (M = 3, SD = 11) condition, both of which were significantly different from zero: t(56) = 6.925, p < .001, d = 0.917 (95% CI: .604 to 1.224), and t(56) = 2.097, p = .041, d = 0.278 (95% CI: .012 to .541), respectively, though the Bayes factor only provided strong evidence for response repetitions (BF_10_ = 2.587 × 10^6^) and was inconclusive for response changes (BF_10_ = 1.099).

## Discussion

Two clear patterns emerged from the data. Deviant sounds slowed response repetitions relative to standard sounds (a deviance distraction effect) regardless of how many response alternatives were available. At the same time, deviant sounds speeded responses on response-change trials relative to standard sounds (a facilitation effect) when the alternative response was uniquely determined: facilitation was significantly stronger in the 2-AFC condition than in the 4-AFC condition. These two effects are dissociable. The deviance distraction effect was immune to the manipulation that selectively attenuated the facilitation effect, establishing that the impact of deviant sounds on responses does not reflect the operation of a unitary phenomenon but that of at least two functionally independent mechanisms.

The finding that deviant sounds both hinder response repetition and facilitate response change replicates and extends García-López and Parmentier [42], who reported a comparable dissociation using a 2-AFC task with controlled response sequence proportions. The present results go further by demonstrating that these two effects have different relationships with response uncertainty. The invariance of the deviance distraction effect across set sizes is consistent with a response-specific inhibitory mechanism: when the environment signals an unexpected change, the previously executed response is suppressed independently of how many alternatives exist. This parallels the global motor inhibition documented by Wessel and Aron [8,52], who showed that unexpected events trigger rapid, non-selective inhibition through cortico-subthalamic pathways. The present data suggest, however, that response inhibition may include a later, response-specific component: it targets the prior response preferentially, since it is the deviance distraction for response repetitions (not a general slowing of all responses) that is invariant across set sizes. The scaling of the facilitation effect with response certainty, by contrast, implies an additional, more context-sensitive process. When only one alternative exists (2-AFC), the signal to abandon the prior response translates directly and efficiently into selection of the sole remaining option. When three alternatives compete (4-AFC), the same signal must be distributed across multiple candidates, diluting the activation of any single one and attenuating the observed speeding.

That response times vary with response set size is well established [60,61], with responses generally faster as the number of alternatives decreases. While Hick attributed this to stimulus uncertainty, later work suggested that response uncertainty plays the larger role [73]. In our study, both conditions used the same four visual stimulus locations, and therefore only response uncertainty differed. Our complementary analysis within the 2-AFC condition (where response repetitions could be accompanied by either stimulus repetition or stimulus change) revealed that deviance distraction was significantly greater when both stimulus and response repeated (d = 0.92) than when only the response repeated (d = 0.28). This closely mirrors Roeber et al.’s [44] finding of larger deviance distraction for identical transitions than for equivalent transitions, indicating that both stimulus-level and response-level information contribute to the inhibitory component of deviance distraction.

Roeber et al. demonstrated, using a 4-to-2 stimulus-response mapping (rare 12.5% vs. frequent 50% pitch changes), that deviance distraction effects varied with the type of sequential transition: deviance distraction was largest when both stimulus and response repeated (identical syllable transitions), intermediate when only the response repeated (equivalent transitions), and absent or reversed for complete changes. They also showed that rare deviants produced larger deviance distraction for response repetitions and a smaller response-change benefit than frequent pitch changes, leading them to attribute both effects to a ‘motor bias toward change.’ Our data support a similar broad conclusion (namely that deviance distraction is greater for repetitions than changes) and extend it by establishing that the two components of this pattern are mechanistically separable. Importantly, in Roeber et al.’s design, manipulating deviant probability necessarily affected both response types simultaneously, which does not allow us to determine whether their differential sensitivity to rarity reflected a single modulated process or two independent mechanisms. Our response set size manipulation circumvents this problem: by holding deviance probability constant while varying response uncertainty, we imposed a manipulation that, under independent mechanisms, should selectively affect switch facilitation while leaving repetition inhibition intact. The dissociation we observed is precisely this pattern. It is also instructive to consider why Roeber et al. [44] found a smaller response-change benefit for rare than for frequent pitch changes — a finding that might appear to be in tension with our demonstration of robust facilitation in the rare-deviant condition. The resolution lies in the comparison being made. In Roeber et al., the rare-deviant condition was compared against an equiprobable-change condition in which pitch changes occurred on 50% of trials. In such a high-frequency change context, contingency-learning processes likely build a strong, standing expectation for change, generating a large switch benefit through a different route than acute deviance detection. Our design includes no such comparison condition; deviants are always rare, and we compare their effects against a standard-sound baseline within the same sequence. The facilitation we observe is therefore best understood as the acute switch-facilitation produced by rare deviance per se, not as contingency-learning-driven switch preparation. Seen in this light, the two studies are complementary: Roeber et al. establish that rarity matters for both components of the effect; the present data establish that the two components have different computational dependencies.

While our study is firmly situated within the oddball and deviance distraction literature, two theoretical accounts from the sequential effects literature offer partially overlapping but ultimately distinguishable predictions for the present pattern. According to Event-File Theory [74–77], the cognitive system automatically integrates episode features (including stimulus properties and motor responses) into unified “event files”. When a stimulus feature repeats on a subsequent trial, it reactivates the associated event file, facilitating the required response if the retrieved response matches, and producing conflict if it does not, either through top-down resolution [75] or through the cost of unbinding the feature from its prior association [78]. Signaling Theory [79], by contrast, posits that the system codes each stimulus as a repetition or a change relative to the immediately preceding trial and uses this binary signal heuristically to guide response selection: stimulus repetition biases the system toward response repetition; stimulus change biases it toward response switching. Both theories can account for the deviance distraction effect on response repetitions. Within Event-File Theory, the deviant sound, having changed from the prior standard, conflicts with the standard-sound feature bound in the previous event file, generating interference that is independent of how many alternative responses exist, consistent with the invariant deviance distraction effect. Within Signaling Theory, the deviant sound signals ‘change,’ placing an inhibitory tag on the prior response regardless of set size (equally consistent with our data). The two theories diverge, however, when applied to the facilitation of response changes. Event-File Theory predicts that a deviant sound combined with a response change forms a new event file without conflict, yielding faster RTs relative to standard-sound change trials, but this prediction is entirely independent of response set size, since event-file formation operates at the level of specific stimulus-response bindings rather than the structure of the broader response space. Signaling Theory, by contrast, predicts the set-size modulation directly: a ‘switch away from R1’ signal is maximally informative when only one alternative exists (pointing unambiguously to R2), and progressively less informative as the number of alternatives increases (distributing activation across R2, R3, R4). The differential sensitivity of facilitation to response uncertainty is therefore more naturally accommodated by Signaling Theory than by Event-File Theory.

One interesting consideration is whether our effects reflect deviant rarity (surprise and prediction violation) or simply the perceptual change from one sound to another. Within the oddball literature, the distinction is not straightforward since surprise follows from the violation of predictions, whether this violation reflects an unexpected change in sensory input (expecting one sound and being presented with another; [4,29,30,32]), or a more abstract violation of a rule [31,32,80]. However, studies examining partial repetition costs have shown that behavioral performance can be affected by mere perceptual changes from one stimulus to another in the absence of surprise [74,77,79,81]: responses are fastest when both stimulus features and responses either repeat or change together, and slowest when only one repeats while the other changes. As in most oddball paradigms, deviant sounds in our design necessarily coincided with a stimulus change from the preceding sound, while standard sounds predominantly constituted stimulus repetitions (standards immediately following a deviant were excluded from analysis). This means that the effect of surprise induced by the deviant sounds in our experiment may potentially have been mitigated by the effect of a mere local stimulus change. While our design was not aimed at addressing this specific issue, it is worth noting that Roeber et al.’s [44] inclusion of an equiprobable-change condition (50% pitch changes) directly addressed this aspect and found that rarity matters beyond mere stimulus change: rare deviants (12.5%) produced larger deviance distraction for response repetitions and smaller facilitation for response changes compared to frequent changes. This established that deviance distraction cannot be reduced to sequential effects alone and that the surprise component engages specialized processing. While within the oddball literature sensory change is not regarded as competing with surprise but rather as a primary mechanism through which surprise emerges (through violations of sensory predictions or of more abstract rules), future work should separate experimentally the impact of surprise from that of mere local stimulus changes (for example, combining probability manipulations, as in Roeber et al. [44], or introducing some level of sensory change among standard trials). Since our study did not include a condition aimed at disentangling the possible role of stimulus change from that of surprise, our conclusions should be taken as tentative.

Finally, one aspect of method deserves mention as a possible limitation. Our 2-AFC and 4-AFC conditions differed not only in the number of response alternatives but also in stimulus-response mapping complexity: the 2-AFC condition employed a 2-to-1 mapping (two stimulus locations per response key), while the 4-AFC condition used a 1-to-1 mapping. Although the invariance of repetition inhibition across conditions argues against mapping complexity as the primary driver of our results, it is true that participants in the 2-AFC condition had to maintain a slightly more complex stimulus-response mapping representation (2-to-1) compared to participants in the 4-AFC condition (1-to-1). We would argue that the mapping was unlikely to have been highly demanding. Further, we note that response accuracy did not differ between these two conditions (numerically, it was in fact slightly lower in the 4-AFC condition than in the 2-AFC). Hence, it is not clear to us that a 2-AFC design using only two stimulus locations (instead of four) would have constituted a better choice. Such an alternative would, in fact, have made the task distinct from the perspective of the stimulus presentation and would have confounded stimulus set size and response set size, which we purposefully aimed to avoid.

In conclusion, the present findings reveal that deviant sounds exert dual and dissociable effects on action control: they inhibit repetition of the prior response in a manner independent of response uncertainty, and they facilitate selection of an alternative response in a manner that scales inversely with the number of available alternatives. By using a response set size manipulation while holding deviance probability constant and varying the structure of the response space, we were able to empirically separate effects that previous designs conflated. These results challenge a unitary conception of deviance distraction and align more naturally with a dual-process framework in which a relatively automatic inhibitory mechanism operates in parallel with a more context-sensitive facilitative one. They also underscore the adaptive character of orienting mechanisms: rather than simply interrupting behavior, unexpected sounds appear to simultaneously suppress what was being done and channel the system toward what could be done next. Future work that combines probability manipulations with response set size manipulations, aiming to separate sensory change from surprise, or probing the neural time course of the deviance distraction and facilitation effects via EEG, would provide a useful further test of this dual-process account.
